# Bac2Feature: an easy-to-use interface to predict prokaryotic traits from 16S rRNA gene sequences

**DOI:** 10.1093/bioadv/vbaf136

**Published:** 2025-06-14

**Authors:** Masaki Fujiyoshi, Takao K Suzuki, Chikara Furusawa, Motomu Matsui

**Affiliations:** Department of Integrated Biosciences, Graduate School of Frontier Sciences, The University of Tokyo, Kashiwa, Chiba 277-8561, Japan; Department of Integrated Biosciences, Graduate School of Frontier Sciences, The University of Tokyo, Kashiwa, Chiba 277-8561, Japan; Department of Biochemistry and Systems Biomedicine, Juntendo University Graduate School of Medicine, 2-2-1, Hongo, Bunkyo-ku, Tokyo 113-8421, Japan; Universal Biology Institute, Graduate School of Science, The University of Tokyo, Tokyo 113-0033, Japan; Center for Biosystems Dynamics Research (BDR), RIKEN, Kobe, Hyogo 650-0047, Japan; Department of Integrated Biosciences, Graduate School of Frontier Sciences, The University of Tokyo, Kashiwa, Chiba 277-8561, Japan; Institute for Chemical Research, Kyoto University, Uji, Kyoto 611-0011, Japan

## Abstract

**Motivation:**

Annotating physiological, morphological, or genomic features to diverse bacterial and archaeal species is essential for the trait-based interpretations of complex microbiomes in community ecology. One promising way for high-throughput trait annotation is taxonomic marker gene-based trait prediction, where traits are predicted from 16S rRNA gene sequences based on their homology, taxonomy, or phylogeny. Many tools have been developed using different prediction methods and trait datasets; however, this situation lacks a unified framework of different prediction methods and their systematic evaluation, resulting in low accessibility to preferred methods.

**Results:**

Here, we proposed Bac2Feature, an easy-to-use web tool that integrates different prediction methods in standardized trait dataset and systematically evaluated the prediction performance. We found that the prediction method based on 16S rRNA gene phylogenetic tree displayed superior prediction accuracy than that of other methods. The prediction accuracy of each trait was strongly associated with its phylogenetic signal. Furthermore, we set prediction thresholds for phylogenetic distance to avoid spurious trait prediction. Applied to the infant gut microbiome, Bac2Feature could reproduce trait-based shifts during primary succession. Our unified framework widens access to the trait-based approach and its applications to other fields, such as medical research.

**Availability and implementation:**

Bac2Feature is available at https://github.com/fuyo780/Bac2Feature (command line interface) and https://www.genome.jp/bac2feature/ (web interface).

## 1 Introduction

The trait-based approach is increasingly utilized in microbial ecology to elucidate the mechanisms driving community function and assembly (Lajoie and Kembel 2019). Integrated with amplicon sequencing, it emphasizes ecologically relevant traits of microbiome members over their taxonomy. Such integration has identified microbial community traits and functions (Bewick *et al.* 2019, Mise and Iwasaki 2020) and established generalized patterns in their assembly (Louca *et al.* 2016, Ortiz-Álvarez *et al.* 2018, Abreu *et al.* 2023). A critical step in the trait-based analysis of amplicon sequencing data is annotating prokaryotic traits, linking specific characteristics to microbiome members. Given that high-throughput trait assays are generally confined to several phenotypes observable via microscopy (Kritikos *et al.* 2017), a scalable alternative is predicting prokaryotic traits from taxonomic genes acquired through amplicon sequencing (Martiny *et al.* 2015).

Numerous trait prediction techniques have been developed to infer traits using taxonomic genes (Djemiel *et al.* 2022), particularly the 16S rRNA gene in prokaryotes. Although these tools predict prokaryotic traits from 16S rRNA gene sequences, they employ various methodologies for trait prediction. These methodologies are classified into three representative approaches: homology-, taxonomy-, and phylogeny-based (Fig. 1). Each method infers phylogenetic information from 16S rRNA gene sequences through distinct processes: homology search, taxonomic classification, and phylogenetic placement. These methods have been employed in numerous studies (Okuda *et al.* 2012, Cébron *et al.* 2021, Abreu *et al.* 2023), with tools such as Tax4Fun2 (Wemheuer *et al.* 2020) for homology-based prediction, FAPROTAX (Louca *et al.* 2016) for taxonomy-based prediction, and PICRUSt2 (Douglas *et al.* 2020) for phylogeny-based prediction. However, the accessibility to preferred methods is hindered by an array of separated prediction techniques. Moreover, the reliability of trait predictions using 16S rRNA gene sequences are frequently criticized for inaccuracies (Sun *et al.* 2020, Matchado *et al.* 2023), requiring systematic evaluations of prediction accuracy.

**Figure 1.**
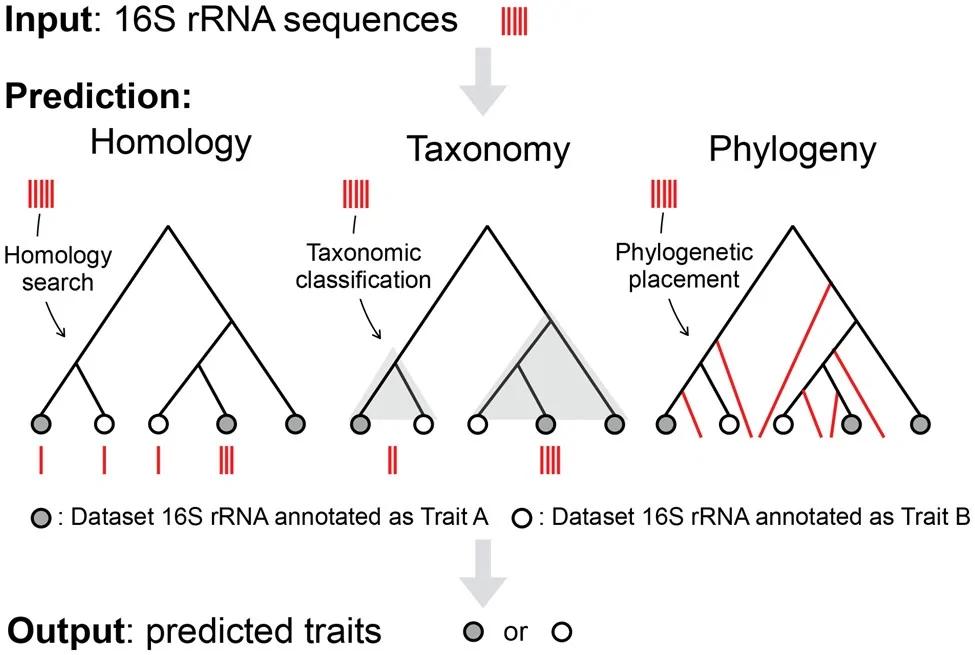
Conceptual description of the three prediction approaches integrated in Bac2Feature. Bac2Feature inputs 16S rRNA gene sequences (top) and generates predictions of prokaryotic traits (bottom) through three prediction methods: homology-, taxonomy-, and phylogeny-based prediction. The input 16S rRNA gene sequences are matched with reference 16S rRNA gene sequences according to their similarities (left), taxonomic classifications (middle), and phylogenetic relationships (right). The shaded areas in the middle tree represent taxonomic groups, while the red lines in the right tree represent the positions of the input 16S rRNA gene sequences as determined by phylogenetic placement. Each dot at the tips represents traits of prokaryotic species from the reference dataset.

Here, we developed Bac2Feature, an easy-to-use interface that integrates three representative trait prediction methods and provides a systematic evaluation of their performance. We assessed the accuracy and computational speed of homology-, taxonomy-, and phylogeny-based trait prediction using a standardized trait dataset encompassing 11 continuous and 16 categorical traits. We further investigated associations between trait prediction accuracy and phylogenetic signals. Additionally, we analysed trait autocorrelations across phylogenetic distances and established prediction thresholds for phylogenetic distances. Finally, we applied Bac2Feature to 16S rRNA gene amplicon sequencing data of the gut microbiome, demonstrating its ability to effectively reproduce trait-based patterns in primary succession and its suitability for real-world applications.

## 2 Methods

### 2.1 Datasets

#### 2.1.1 Trait data of prokaryotes

Trait data of 14 893 prokaryotes were downloaded from the database (https://github.com/bacteria-archaea-traits/bacteria-archaea-traits/blob/master/output/condensed_species_NCBI.csv, downloaded 6 June 2024) in a previous study (Madin *et al.* 2020). To avoid bias toward well-studied species, such as *Escherichia coli*, we used trait data grouped by species, not strain-level data. The original dataset comprised 11 continuous and 10 categorical traits. It contained five genomic traits (e.g. genome size), 11 phenotypic traits (e.g. Gram staining), and five environmental preferences (e.g. optimum temperature). We excluded three phenotypic traits (isolation source, carbon substrates, and pathways) from our analysis because these traits had diverse classes. The complete lists of traits and their data types in the original dataset are summarized in Table 1.

**Table 1.** The description of the trait data obtained from a previous study (Madin *et al.* 2020).

Trait	Our study	Data types	Trait types	Preprocessing
Genome size	Used	Continuous	Genomic trait	Not
GC content	Used	Continuous	Genomic trait	Not
Coding genes	Used	Continuous	Genomic trait	Not
rRNA16S genes	Used	Continuous	Genomic trait	Not
tRNA genes	Used	Continuous	Genomic trait	Not
Cell diameter	Used	Continuous	Phenotypic trait	Log scaling
Cell length	Used	Continuous	Phenotypic trait	Log scaling
Doubling time	Used	Continuous	Phenotypic trait	Log scaling
Optimum pH	Used	Continuous	Environmental preference	Not
Optimum temperature	Used	Continuous	Environmental preference	Not
Growth temperature	Used	Continuous	Environmental preference	Not
Isolation source	Not used	Categorical (72)	Environmental preference	–
Gram stain	Used	Categorical (2)	Phenotypic trait	Not
Respiration (Metabolism)	Used	Categorical (6)	Phenotypic trait	Character coding (6 to 3) and conversion to binomial traits
Pathways	Not used	Categorical (18)	Phenotypic trait	–
Carbon substrates	Not used	Categorical (69)	Phenotypic trait	–
Sporulation	Used	Categorical (2)	Phenotypic trait	Not
Motility	Used	Categorical (5)	Phenotypic trait	Character coding (5 to 2)
Cell shape	Used	Categorical (14)	Phenotypic trait	Removing minor classes(14 to 6) and conversion to binomial traits
Salinity range	Used	Categorical (7)	Environmental preference	Character coding (7 to 4) and removing minor classes (4 to >2)
Temperature range	Used	Categorical (7)	Environmental preference	Character coding (7 to 3) and conversion to binomial traits

The number in a bracket for each categorical trait indicates the number of its classes. The classification of the trait types follows that of the previous study.

#### 2.1.2 Concatenation of the trait data to 16S rRNA gene sequences

Full-length 16S rRNA gene sequences of prokaryotes in the trait dataset were obtained from the SILVA SSU Ref NR99 database version 138.1 (Yilmaz *et al.* 2014, downloaded 6 June 2024). For each prokaryote, one 16S rRNA gene sequence was extracted based on two alignment quality metrics in the SILVA database (Pruesse *et al.* 2007). The “align_quality_slv” score was firstly used to select sequences, followed by the “align_bp_score_slv” score for sequences with equal initial values. Then, we combined the selected 16S rRNA gene sequences with the trait data described above using their NCBI taxonomy ID, leaving 13 166 out of 14 893 species.

#### 2.1.3 Preprocessing the trait data

We preprocessed three continuous and five categorical trait datasets from the original dataset (Table 1). For continuous traits, we transformed cell diameter, length, and doubling time into a common logarithmic scale because this transformation is commonly used to study cell size and growth rates (Westoby *et al.* 2021, Secaira-Morocho *et al.* 2024). For categorical traits, three preprocessing steps were performed. First, we reorganized the classes of the four categorical traits to ensure mutual exclusivity among classes. Complete rules of character coding are provided in Supplementary Table S1, available as supplementary data at *Bioinformatics Advances* online. Secondly, we removed minor classes that constituted <1% of the data for each categorical trait, excluding two classes in the salinity range and eight in cell shape (Supplementary Table S2, available as supplementary data at *Bioinformatics Advances* online). Finally, we divided the classes of each categorical trait into binomial traits, yielding 12 binomial traits from three multi-categorical traits. The distribution and number of data records of the reference trait data after the preprocessing, used in the following analysis, are shown in Supplementary Fig. S1, available as supplementary data at *Bioinformatics Advances* online and Supplementary Table S3, available as supplementary data at *Bioinformatics Advances* online, respectively.

#### 2.1.4 Phylogenetic tree and multiple sequence alignment of the 16S rRNA gene

We downloaded the Living Tree Project’s phylogenetic tree from the SILVA SSU Ref NR99 database version 138.1 (Yilmaz *et al.* 2014, downloaded 6 June 2024). Nodes of prokaryotes not included in the reference trait data were pruned using the TreeNode.prune function in ete3 toolkit 3.1.1 (Huerta-Cepas *et al.* 2016) with the option “preserve_branch_length = True”. Starting with the pruned phylogeny, branch lengths of the 16S rRNA gene phylogenetic tree were optimized using RAxML-NG version 1.1 (Kozlov *et al.* 2019) with the option “—evaluate—model GTR+G+F—blmin 0.0001—blmax 1”. We also downloaded the multiple sequence alignment of the 16S rRNA gene sequences from the SILVA database (downloaded 6 June 2024) and extracted sequences of prokaryotes in the reference trait data. The multiple sequence alignment was subsequently trimmed using trimAl version 1.4 (Capella-Gutiérrez *et al.* 2009) with the option “-gt 0.10”.

### 2.2 Prediction of prokaryotic traits with Bac2Feature

We implemented three methods for predicting prokaryotic traits using 16S rRNA gene sequences: homology-, taxonomy-, and phylogeny-based prediction. These methods employ different approaches for inferring phylogenetic information, each using homology search, taxonomic classification, and phylogenetic placement.

#### 2.2.1 Homology-based prediction

Homology-based prediction used an input 16S rRNA gene sequence in homology search against the 16S rRNA gene sequences combined with the reference trait data using BLAST version 2.12.0+ (Camacho *et al.* 2009). For each trait, species with the highest similarity—percentage identity in aligned regions—in the 16S rRNA gene sequence were identified among species with known traits whose trait was assigned to the input 16S rRNA gene sequence. To evaluate possible variations with the homology-based prediction, we tested three homology search algorithms: BLAST+ local alignment, VSEARCH global alignment version 2.22.1 (Rognes *et al.* 2016), and SortMeRNA rRNA-specific alignment version 2.0 (Kopylova *et al.* 2012). These differences did not lead to significant differences in the prediction accuracy for most of continuous and categorical traits (Supplementary Fig. S2, available as supplementary data at *Bioinformatics Advances* online). Therefore, we selected BLAST+ for the homology-based prediction because of its realistic computation time for local alignment and its expandability by being applicable to more than just rRNA genes.

#### 2.2.2 Taxonomy-based prediction

Taxonomy-based prediction used an input 16S rRNA gene sequence in taxonomic classification using the q2-naïve-bayes classifier version 2023.5.0 (Bokulich *et al.* 2018), trained with the reference dataset using the function “qiime feature-classfier fit-classifier-naive-bayes”. The 16S rRNA gene sequence was matched to the average or majority of traits in the classified taxonomic clade for continuous and categorical traits, respectively. If the input sequence was not classified in some taxonomic rank, it was iteratively matched to a higher taxonomic rank, following previous methodologies (Cébron *et al.* 2021, Wilhelm *et al.* 2023). To evaluate possible variations with the taxonomy-based prediction, we tested three taxonomic classifiers in QIIME 2 version 2023.5.0: a k-mer-based naïve Bayes classifier and alignment-based consensus classifiers using BLAST+ or VSEARCH, all of which have been reported to perform comparably or better than other classification methods (Bokulich *et al.* 2018). The prediction using the naïve Bayes classifier outperformed those using the alignment-based classifiers for both continuous and categorical traits (Supplementary Fig. S3, available as supplementary data at *Bioinformatics Advances* online). Therefore, we selected the naïve Bayes classifier for the taxonomy-based prediction in this study.

#### 2.2.3 Phylogeny-based prediction

Phylogeny-based prediction used an input 16S rRNA gene sequence for phylogenetic placement on the 16S rRNA gene phylogenetic tree combined with the reference trait data. Phylogenetic placement was performed using the “place_seqs.py” function in PICRUSt2 version 2.5.2 (Douglas *et al.* 2020), a wrapper of EPA-ng version 0.3.8 (Huerta-Cepas *et al.* 2016). Traits were then predicted using the *castor* package version 1.7.2 in R (Louca and Doebeli 2018) with the “hsp_squared_change_parsimony” function applied to continuous traits and the “hsp_max_parsimony” function to categorical traits. To evaluate possible variations with the phylogeny-based prediction, we tested several hidden state prediction (HSP) algorithms for inferring traits from the 16S rRNA gene phylogenetic tree. Following to the methodology of a previous study (Gao and Wu 2023), we compared three HSP algorithms for continuous traits: weighted squared-change parsimony, subtree averaging, and phylogenetic independent contrast. For categorical traits, we compared five HSP algorithms for categorical traits: empirical probability, Mk-symmetrical-model, Mk-all-rate-differential-model, and maximum parsimony with or without considering edge lengths. All HSP algorithms were implemented in the castor package version 1.7.2 in R (Louca and Doebeli 2018). Weighted squared-change parsimony showed the highest accuracy for predicting the continuous traits (Supplementary Fig. S4a, available as supplementary data at *Bioinformatics Advances* online). Therefore, we selected weighted squared-changed parsimony in our study. For the categorical traits, no clear differences in accuracy were observed among the four HSP algorithms excluding empirical probability (Supplementary Fig. S4b, available as supplementary data at *Bioinformatics Advances* online). Therefore, we selected maximum parsimony with branch length consideration in our study for its simplicity.

### 2.3 Evaluation of prediction accuracy by cross-validation

To evaluate the prediction accuracy of the three aforementioned methods, we performed 10-fold cross-validation. We randomly split the reference dataset, which consisted of trait data, 16S rRNA gene sequences, multiple sequence alignment, and phylogenetic tree. For each validation test, species traits in one part of the dataset were predicted using the three methods based on the remaining dataset. Prediction accuracy for continuous traits was evaluated using the Pearson correlation coefficient between the predicted and actual traits. Prediction accuracy for categorical traits was evaluated using the Matthews correlation coefficient because it is reportedly well-suited for evaluating prediction accuracy in unbalanced trait datasets (Koblitz *et al.* 2025). Statistical differences in accuracy among the three methods were tested using the “friedmanchisquare” function (Friedman’s test) in the *scipy* package version 1.10.0 for Python (Virtanen *et al.* 2020), with false discovery rate (FDR)-corrected *P*-values (*q *= 0.05) from Benjamini–Hochberg’s method. The ranks of the three methods were determined from statistical differences derived from the “calc_wilconxon_signed” function in the *scipy* package version 1.10.0 for Python (Virtanen *et al.* 2020), similarly modified by Benjamini–Hochberg’s method. To consider a more realistic case when the test sequences have no close relatives in the reference, we performed the cross-validation by nonrandom sampling based on taxonomic clades at order, family, and genus levels, namely order-, family-, and genus-out cross-validation. Specifically, we randomly selected 10 clades as test data from the largest 100 clades at each taxonomic rank (e.g. the genus *Streptomyces*). All species within these clades were used as the test data, and the remaining species were used as the reference data. For each taxonomic rank, the nonrandom sampling of 10 clades and their cross-validation were performed 10 times, ensuring the 10 clades were mutually exclusive within the largest 100 clades.

### 2.4 Benchmarking runtime performance of Bac2Feature

We also evaluated the computational speeds of the three aforementioned methods. Non-coding RNA sequences were downloaded from “rnacentral_active.fasta” of RNA central version 21 (RNAcentral Consortium 2021). We extracted 16S rRNA sequences whose source information was “GREENGENES”, type information was “rRNA”, and comment was “rrn”. Subsequently, we randomly selected 16S rRNA sequences as input sequences. The computational time was evaluated using the function “time.perf_counter()” in Python version. 3.8. The benchmark was run on a Red Hat Enterprise Linux 8.7, with 1 CPU core at 2.6 GHz and 40 GB of memory.

### 2.5 Calculation of phylogenetic signals

We calculated phylogenetic signals from the trait data and 16S rRNA gene phylogenetic tree. Prokaryotes missing trait data were pruned from the tree for each trait. Blomberg’s *K* (Blomberg *et al.* 2003) was used for continuous traits and the *D* statistics (Fritz and Purvis 2010) for categorical traits. Blomberg’s *K* and the *D* statistics were calculated using the “multiPhylosignal” function of the *picante* package version 1.8.2 in R (Kembel *et al.* 2010) and the modified “phylo.d” function in the *caper* package version 1.0.3 in R (Orme *et al.* 2025), respectively.

### 2.6 Calculation of phylogenetic distances with moderate trait autocorrelations

To establish prediction thresholds, we calculated trait autocorrelations along with phylogenetic distances in the 16S rRNA gene phylogenetic tree, following a previous study (Louca *et al.* 2018). We binned all species pairs on the tree into one of many phylogenetic distance intervals from 1e−4 to 2 with a width of 0.05 and then randomly picked 1000 species pairs within each bin 100 times. We calculated Pearson’s correlation coefficient between the traits of the species pairs for continuous traits and Matthews’ correlation coefficient for categorical traits. We set the thresholds of phylogenetic distances as trait autocorrelations that were always greater than 0.5.

### 2.7 Calculation of ASV abundance within thresholds in Earth Microbiome Project dataset

To calculate the phylogenetic distances between 16S rRNA gene sequences in the environment and those in the reference dataset, we used 16S rRNA gene amplicon sequencing data across biomes from the Earth Microbiome Project (EMP) dataset (Thompson *et al.* 2017), specifically using a deblur result (downloaded on June 15). Phylogenetic distances between 16S rRNA gene sequences in the environment and those in the reference dataset were calculated using the “hsp_nearest_neighbor” function in the *castor* package version 1.7.2 in R (Louca and Doebeli 2018).

### 2.8 Application to real microbiome datasets

To apply Bac2Feature to real microbiome data, we downloaded three amplicon sequencing datasets from human gut microbiome (Kostic *et al.* 2015), lake water (Mayr *et al.* 2020), and agricultural soil (Zhao *et al.* 2020) environments (Table 2). These datasets were denoised using the DADA2 (Callahan *et al.* 2016) plugin in QIIME2 version 2023.5.0 (Bolyen *et al.* 2019). We then inferred traits from the obtained amplicon sequence variants using the phylogeny-based prediction of Bac2Feature and then filtered the prediction results using the phylogenetic distance thresholds calculated above.

**Table 2.** 16S rRNA gene amplicon sequencing datasets reanalyzed using Bac2Feature.

Sample description	Number of samples	Data availability	Date of download	Reference
Stool of newborn Finnish infants	654	DIABIMMUNE project T1D cohort (https://diabimmune.broadinstitute.org/diabimmune/t1d-cohort)	16 June 2024	Kostic *et al.* (2015)
Saline agricultural soils in north-west China	124	INSD SRP136143	10 October 2023	Zhao *et al.* (2020)
Water sampled from four stratified lakes in Switzerland	132	INSD PRJEB27579	4 September 2023	Mayr *et al.* (2020)

## 3 Results

### 3.1 Performance evaluation of three representative trait prediction methods

To evaluate the performance of trait predictions using the Bac2Feature interface, we conducted 10-fold cross-validation for three distinct methods: homology-, taxonomy-, and phylogeny-based prediction (Fig. 1). The prediction accuracy across 11 continuous traits varied substantially, exhibiting a minimum accuracy of 0.28 for cell diameter and a maximum of 0.95 for GC content (Fig. 2a). Genomic traits such as gene copy number and genome size were predicted relatively accurately compared with phenotypic traits such as cell size and doubling time. Regarding environmental preferences, the prediction accuracy of optimum growth temperature was higher than that of optimum pH. Then, we compared the prediction accuracy of the three methods across the 11 continuous traits. Among the traits, nine exhibited significant differences in accuracy between the three methods (Fig. 2a). The phylogeny-based prediction yielded the highest accuracy, especially for genome size and growth temperature, surpassing those achieved by the homology- and the taxonomy-based predictions. Homology-based prediction consistently showed the lowest accuracy across continuous traits. These results suggest that incorporating phylogenetic tree information explicitly is efficient for predicting continuous traits. Consequently, we conclude that phylogeny-based prediction is the preferred approach for predicting continuous traits.

**Figure 2.**
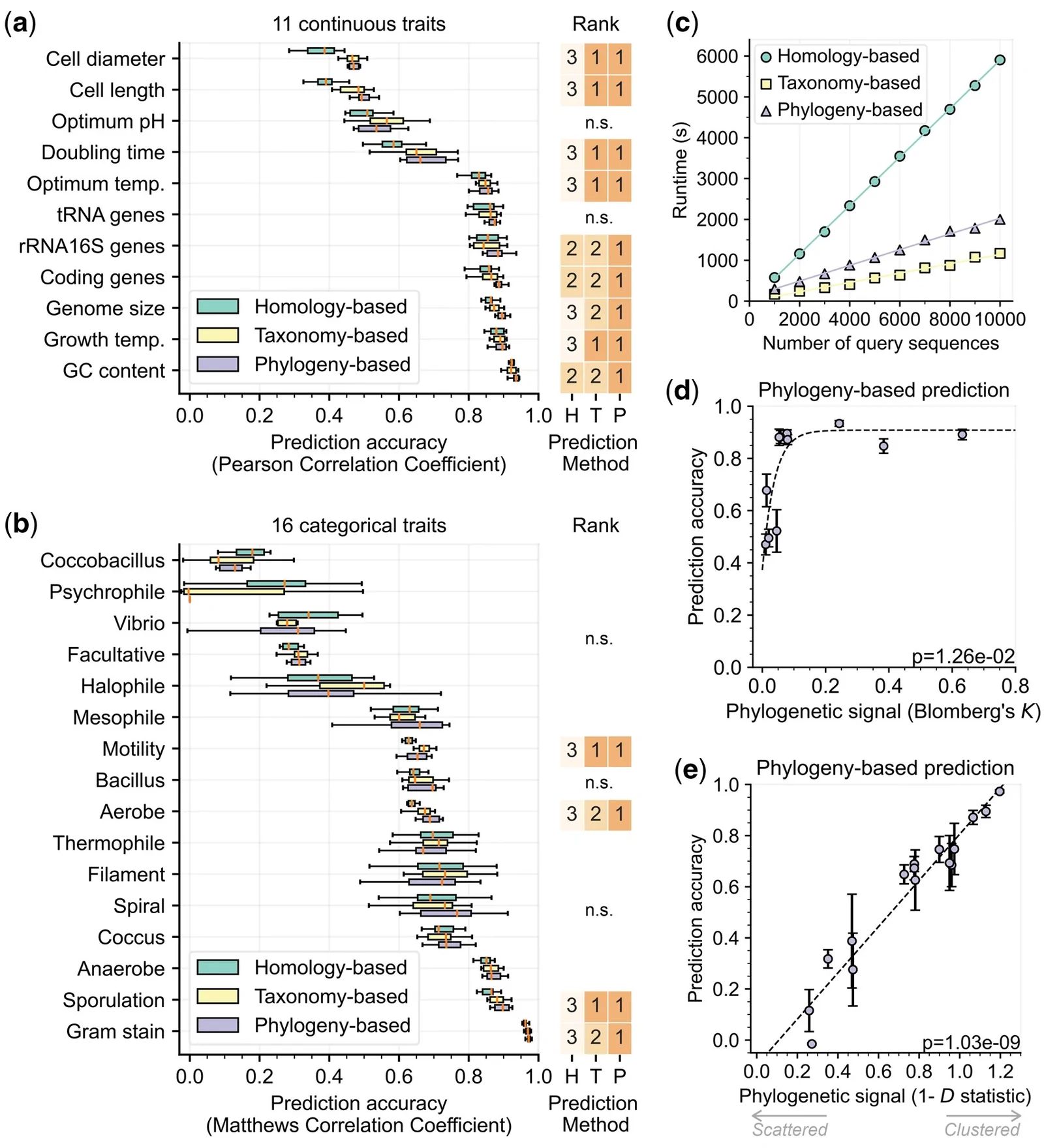
Performance evaluation of the three prediction approaches. (a-b) Prediction accuracy of the homology-, taxonomy-, and phylogeny-based prediction in 10-fold cross-validation for 11 continuous (a) and 16 categorical (b) traits, arranged from lowest to highest average accuracy. Heatmaps on the right show accuracy rankings derived from statistical tests: Friedman’s test for variance analysis and Wilcoxon signed-rank test for determining the rankings. (c) Runtime performance of the three prediction methods, along with the number of query sequences. (d) Relationship between the accuracy of phylogeny-based prediction and a phylogenetic signal, Blomberg’s *K*, in the continuous traits. The dotted line shows the asymptotic regression. (e) Relationship between the accuracy of phylogeny-based prediction a phylogenetic signal, the *D* statistic, in the categorical traits. The dotted line shows the linear regression and asymptotic regression.

We observed similar patterns in the prediction accuracies for the 16 categorical traits (Fig. 2b). The accuracy of the three methods varied considerably across these traits. All methods showed high accuracy for sporulation and Gram staining, whereas they were less accurate for traits such as coccobacillus and psychrophiles. Cell morphologies, such as bacilli, filaments, spirals, and cocci, showed moderate accuracy, whereas coccobacilli and vibrio showed lower accuracy. We found significant differences in the prediction accuracy of the three methods for four of the 16 categorical traits (Fig. 2b). Consistent with the results for continuous traits, the phylogeny-based prediction showed the highest accuracy for aerobes and Gram staining, whereas the homology-based prediction performed the worst for the four categorical traits. We should note that the differences among the three methods were not significant for most categorical traits. This result was due to tradeoff between precision, also specificity (true negative rate), and recall (true positive rate) in the phylogeny- and homology-based predictions (Supplementary Fig. S5, available as supplementary data at *Bioinformatics Advances* online). The phylogeny-based prediction showed superior precision relative to the other methods, whereas the homology-based prediction excelled in recall, leading to a comparable overall accuracy. Therefore, careful selection of prediction methods is essential, depending on whether precision or recall is more important. Because precision is important for the reliability of the predictive outputs of Bac2Feature, we concluded that phylogeny-based prediction should be the primary choice for categorical traits.

In addition to the accuracy of trait predictions, we assessed three practical issues with Bac2Feature. First, we evaluated the runtime performance of the three methods using thousands of 16S rRNA gene sequences (Fig. 2c). We found that the taxonomy-based prediction was the fastest among the methods, with the phylogeny-based prediction also demonstrating competitive performance. Taken together, we found that each of the three methods exhibited their own strengths; consequently, we retained all prediction methods in Bac2Feature. Second, although we performed 10-fold cross-validation using full-length 16S rRNA gene sequences, we also confirmed that the accuracy of the phylogeny-based prediction was not severely compromised by using only variable regions of the 16S rRNA gene sequences (Supplementary Fig. S6, available as supplementary data at *Bioinformatics Advances* online). Finally, we considered a more realistic case when the test sequences have no close relatives in the reference. We performed the cross-validation by nonrandom sampling based on taxonomic clades at the order, family, and genus levels, namely order-, family-, and genus-out cross-validation. As expected, the accuracy of the phylogeny-based prediction decreased from genus-out to order-out cross-validation for both the continuous and categorical traits (Supplementary Fig. S7, available as supplementary data at *Bioinformatics Advances* online). While the prediction accuracy of optimum growth temperature decreased dramatically in the absence of genus-level relatives, genomic traits—including genome size and rRNA copy number—maintained moderate accuracy (Pearson’s *r *> 0.5) even without family-level relatives (Supplementary Fig. S7a, available as supplementary data at *Bioinformatics Advances* online). In contrast, the prediction accuracy for categorical traits such as motility, cell morphology, and temperature range substantially decreased when genus-level relatives were absent (Supplementary Fig. S7b, available as supplementary data at *Bioinformatics Advances* online). These findings highlight the need for future studies to expand trait databases, particularly for traits that depend on the presence of close phylogenetic relatives for accurate prediction.

### 3.2 Strong associations between prediction accuracy and phylogenetic signals

The accuracy of trait prediction using 16S rRNA gene phylogeny has been reported to associate with the trait resemblance between close relatives (Martiny *et al.* 2015, Walkup *et al.* 2023). This prompted us to examine the relationship between prediction accuracy and phylogenetic signals. We investigated the associations between the accuracy of phylogeny-based predictions and phylogenetic signals for 11 continuous (Fig. 2d) and 16 categorical traits (Fig. 2e). We employed two established metrics for phylogenetic signals: Blomberg’s *K* (Blomberg *et al.* 2003) for continuous traits and the *D* statistic (Fritz and Purvis 2010) for categorical traits. We found that the accuracy of the phylogeny-based prediction was positively correlated with the phylogenetic signal for both trait types (Spearman’s *r *= 0.727, *P *= .0112 for continuous traits; Spearman’s *r *= 0.947, *P *= 2.72e−8 for categorical traits). These strong correlations suggest that the trait resemblance between closely related species influences the prediction accuracy of Bac2Feature. For example, the lower prediction accuracy for aerobes and facultatives compared with that for anaerobes may be due to the challenge of distinguishing between aerobes and facultatives in the 16S rRNA gene phylogenetic tree (Supplementary Fig. S8, available as supplementary data at *Bioinformatics Advances* online). It should be noted that the phylogenetic signals did not refer to the evolutionary process or rate (i.e. trait conservatism), but to the phylogenetic patterns (i.e. trait similarity between close relatives), which has often been pointed out (Blomberg *et al.* 2003, Revell *et al.* 2008). Although the prediction accuracies for both continuous and categorical traits were positively correlated with phylogenetic signals as mentioned above, they increased nonlinearly with Blomberg’s *K* (fitted by asymptotic regression model, *P *= .0126, ANOVA test) and linearly with the *D* statistic (fitted by linear regression model, *P *= 1.03e−9, *F* test). The empirical associations observed between prediction accuracy and phylogenetic signals showed qualitatively similar behaviors with those derived from previous theoretical work (Goberna and Verdú 2016), supporting the validity of our analysis. These strong and valid associations between prediction accuracy and phylogenetic signals have prompted us to examine phylogenetic resolution over which traits tended to be conserved, as described below.

### 3.3 Prediction thresholds for phylogenetic distances

Trait prediction using 16S rRNA gene sequences has often been criticized for the inaccuracies of existing tools, particularly those focusing on predicting gene copy numbers (Sun *et al.* 2020, Matchado *et al.* 2023). These inaccuracies often arise when predicting the traits of prokaryotes that are distantly related to those in a reference dataset (Louca *et al.* 2018). To ensure reliable predictions using Bac2Feature, we established thresholds for phylogenetic distances at which trait autocorrelations exceeded a specified level (Fig. 3a, Pearson’s correlation coefficient >0.5 for continuous traits, Matthews’ correlation coefficient >0.5 for categorical traits). Our analysis revealed distinct thresholds for continuous (Fig. 3b) and categorical traits (Fig. 3c). Genomic traits displayed higher thresholds (Fig. 3b) because of greater trait autocorrelations among distantly related species. In contrast, traits such as cell size and optimum pH failed to meet the autocorrelation threshold even among closely related species, leading to their exclusion from the predictive output of Bac2Feature. For categorical traits, Gram staining, sporulation, and anaerobes showed autocorrelations above the threshold, even among distantly related species (Fig. 3c). Unlike the low trait autocorrelations observed for cell size, cell morphologies, including bacillus, coccus, and filament, showed higher autocorrelations than the threshold, indicating that these traits can be inferred from phylogeny at the specified level.

**Figure 3.**
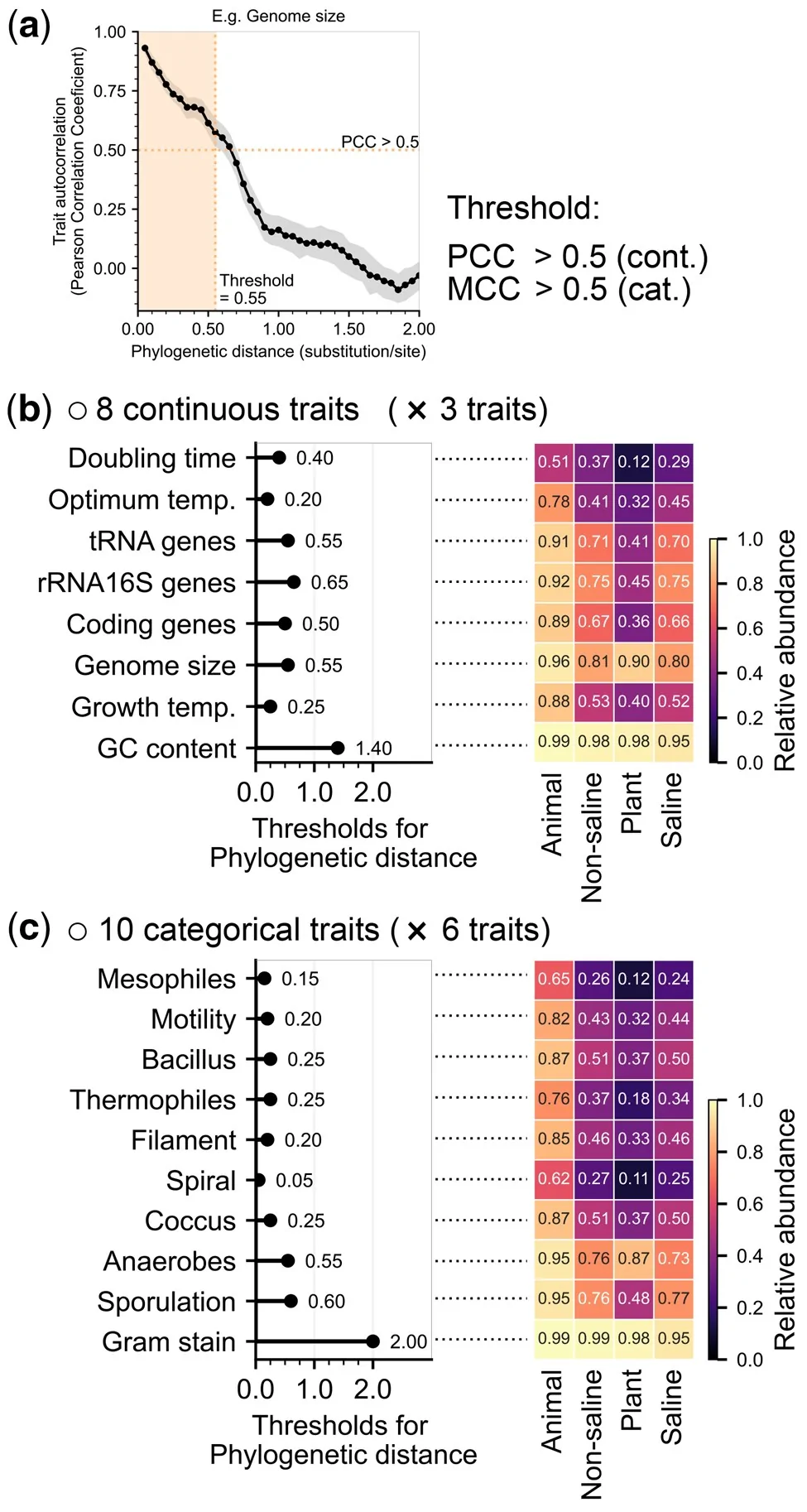
Setting thresholds for phylogenetic distance based on trait autocorrelations. (a) Exemplified determination of prediction thresholds using Pearson autocorrelation of genome size depending on phylogenetic distance between tip pairs. Each dot represents the average of trait autocorrelation, and the shaded area represents the 95% interval of prediction accuracy. (b) Left panel: phylogenetic distances within which the trait autocorrelations of the continuous traits were above the specified threshold, Pearson autocorrelation >0.5. Right panel: mean relative abundance of 16S rRNA gene sequences within the phylogenetic distance for continuous traits in EMP samples. (c) Left panel: phylogenetic distances within which the trait autocorrelations of the categorical traits were above the specified threshold, Mathew’s autocorrelation >0.5. Right panel: mean relative abundance of 16S rRNA gene sequences within the thresholds of phylogenetic distance in samples of Earth Microbiome Project. Categories of biomes follow the EMP Ontology level 2.

To examine whether the analysis of real communities would benefit from the phylogeny-based prediction, we quantified the relative abundance of 16S rRNA gene sequences within established phylogenetic distance thresholds across the EMP dataset (Thompson *et al.* 2017). The highest abundances were observed in samples from environments classified as “Animal” in EMP Ontology Level 2, which includes extensively studied environments such as the human gut microbiome (Fig. 3b and c). Therefore, we strongly recommend that users apply Bac2Feature to animal-associated biomes. In contrast, using the Bac2Feature in less studied environments, such as those associated with plants, should be cautiously approached because of the low abundance of 16S rRNA gene sequences within the defined threshold. These biases are widely observed for other software that depend on reference trait data (Sun *et al.* 2020, Djemiel *et al.* 2022).

### 3.4 Application to real microbiome datasets

#### 3.4.1 Early succesions of human infant gut microbiome

To demonstrate the powerful application of Bac2Feature, we applied it to the 16S rRNA gene amplicon sequencing dataset of human infant gut microbiomes (Kostic *et al.* 2015). A previous study revealed trait-based patterns in the primary succession of the infant gut microbiome by combining 16S rRNA gene amplicon sequencing with 12 prokaryotic traits, including genomic, stress tolerance, and phenotypic traits (Guittar *et al.* 2019). Here, we extended this study by investigating 17 prokaryotic traits, including almost all previously studied traits and additional traits such as doubling time and cell shape. We observed shifts in taxonomic compositions during primary succession (Fig. 4a) and then predicted shifts in the continuous (Fig. 4b) and categorical traits (Fig. 4c) of the entire microbiome using phylogeny-based prediction in Bac2Feature. We first confirmed that Bac2Feature predicted an increase in the optimum growth temperature (Fig. 4b) and anaerobes (Fig. 4c) and a decrease in motility (Fig. 4c), suggesting temporal invasions from the environment into the infant gut microbiome in the early stages of primary succession. These trends are consistent with those of a previous study (Guittar *et al.* 2019), supporting the validity of trait prediction using Bac2Feature.

**Figure 4.**
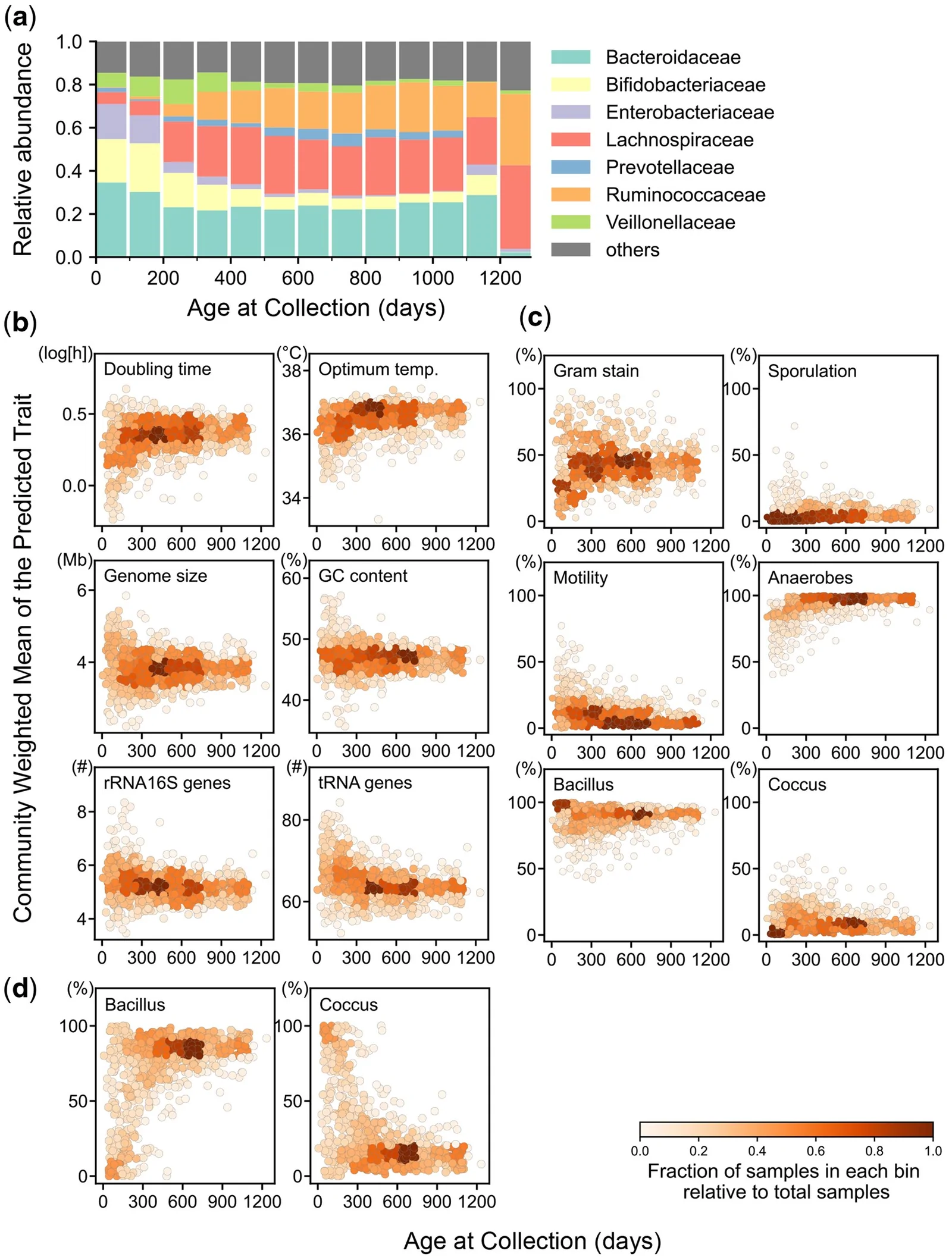
Application to human gut microbiome revealed trait-based community shifts during primary successions. (a) Phylum-level taxonomic compositions of the gut microbiome samples. The samples are sorted by age at the sampling. (b) Relation between age and community-weighted means of continuous traits, (c) relation between age and community-weighted means of categorical traits, (d) relation between age and community-weighted means of cell shapes within *Firmicutes*.

Bac2Feature predicted an increase in the community-weighted mean of doubling time during primary succession (Fig. 4b), which was newly investigated in our study. This increase in doubling time suggests the initial presence of fast-growing prokaryotes in the infant gut microbiome at an early age (Fig. 4b). Although the increase in doubling time aligned with the decrease in rRNA gene copy number reported in a previous study (Guittar *et al.* 2019), our analysis clearly predicted the initial presence of fast-growing species and their subsequent reduction in primary succession. We also newly predicted a decrease in tRNA gene copy numbers during primary succession (Fig. 4b). This decrease supports the shifts in growth rate since tRNA gene copy numbers reportedly positively correlate with codon usage bias in prokaryotes (Wei *et al.* 2019). Taken together, these results suggested that the wide variety of traits predicted by Bac2Feature improved the reliability of trait predictions.

Changes in community-weighted means of genomic (Fig. 4b) and categorical traits, such as Gram staining and sporulation (Fig. 4c), indicated high functional variabilities in the early stages of primary succession, which tended to decrease as age increased. Trait variability in the early stages was partly due to taxonomic variability in the samples at those ages (Fig. 4a). The decrease in these functional variabilities until 1000 days suggested that the infant gut microbiome converged to some state during primary succession. In addition to the trait shifts in the total microbiomes, we also investigated trait-based shifts during primary succession within the dominant phylum *Firmicutes*, *Actinobacteria*, *Bacteroidota*, and *Proteobacteria*. Shifts in the predicted traits were largely driven by shifts within *Firmicutes*. Moreover, we found clear shifts in cell shape within *Firmicutes*, where cocci were replaced with bacillus as age increased (Fig. 4d).

#### 3.4.2 Oxygen gradient of lake water microbiome and salinity gradient of soil microbiome

To explore the relevance of Bac2Feature, we applied it to the 16S rRNA gene amplicon sequencing dataset sampled from stratified lakes in Switzerland (Mayr *et al.* 2020). We predicted the relative fractions of oxygen-tolerant species in the lake water microbiome by using the phylogeny-based prediction, as the anaerobes were predicted well from the 16S rRNA gene sequences (Fig. 2b, MCC > 0.8). We confirmed that the fractions of oxygen-tolerant species in the microbiome increased in elevated oxygen concentrations in the lake water (Fig. 5a, Spearman’s *r *= 0.290, *P *= 7.31e−4). The microbiome at the high oxygen concentrations (>200 µmol/l) had the high fraction of oxygen-tolerant species. In contrast, the microbiome in the water with no-detectable oxygen concentrations (<1 µmol/l) had variable fractions of oxygen-tolerant species, suggesting the occasional persistence of anoxic bacteria in oxygen-deficient lake water columns.

**Figure 5.**
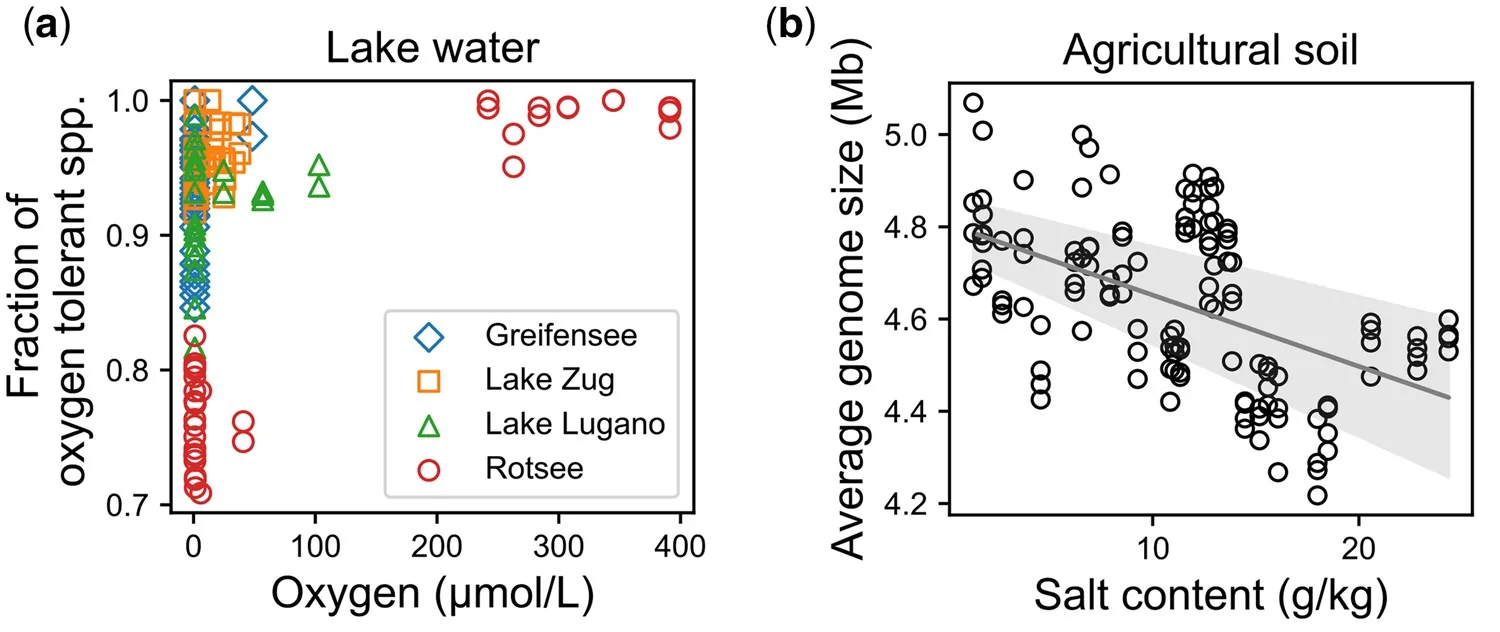
Application to lake water and agricultural microbiome revealed trait-based community shifts along with environmental gradients. (a) Relationship between the fractions of oxygen-tolerant species in the microbiome and dissolved oxygen concentration in lake water. (b) Relationship between the community-weighted means of genome size in the microbiome and salinity in agricultural soil.

We also applied Bac2Feature to saline soil microbiome, which was sampled from Chinese agricultural soil (Zhao *et al.* 2020). We found that the community-weighted mean of the genome size decreased along with the salinity gradient (Fig. 5b, Pearson’s *r *= −0.514, *P *= 1.01e−9), suggesting that smaller genome size might be favorable in high salinity soil. This is consistent with the idea of prokaryotic genome streamlining in stressful environments such as high temperature (Sabath *et al.* 2013) and low resource availability (Chen *et al.* 2021). These case studies of trait-based analysis in the human gut, lake, and soil microbiome showed the effectiveness of Bac2Feature in various environments.

## 4 Discussion

In this study, we developed Bac2Feature, a unified interface that integrates methodologies for predicting prokaryotic traits from 16S rRNA gene sequences and conducted systematic evaluations using a standardized trait dataset. Cross-validation showed that phylogenetic tree-based prediction outperformed the other methods in prediction accuracy, though the other methods excelled in recall (true positive rate) or computational speed. By setting phylogenetic distance thresholds based on trait autocorrelations, eight continuous and 10 categorical traits remained for application to amplicon sequencing data, particularly from animal-associated environments. Our applications of Bac2Feature to the real microbiome datasets, demonstrated the validity, efficiency, and accessibility of this interface.

Several limitations are associated with predicting prokaryotic traits using Bac2Feature. One limitation is its poor prediction accuracy for several traits such as cell size, optimum pH, coccobacillus morphology, and facultative respiration (Fig. 2a and b). Difficulties in predicting these traits may stem from their low heritability (Ramoneda *et al.* 2023, Secaira-Morocho *et al.* 2024) and similarity to other states (Koblitz *et al.* 2025). These traits need to be quantified in high-throughput assays to study these distributions in the environment. Another limitation is that trait prediction using Bac2Feature is biased toward animal environments (Fig. 3). Although limited representation of current prokaryotic trait databases constrains the effectiveness of Bac2Feature, future expansion of the databases would enhance its application scope.

Compared with existing tools for predicting prokaryotic traits from 16S rRNA gene sequences, Bac2Feature proposes an integrated framework using implementations previously employed in separate studies (Louca *et al.* 2016, Douglas *et al.* 2020, Wemheuer *et al.* 2020). Through a standardized evaluation of prediction accuracy, Bac2Feature provides a basis for selecting preferred methods. Moreover, Bac2Feature predicts various traits, including quantitative genomic and qualitative phenotypic traits, whereas most existing tools focus solely on functional gene copy numbers (Okuda *et al.* 2012, Douglas *et al.* 2020, Wemheuer *et al.* 2020). Additionally, as previous studies have reported uncertainty in trait predictions by existing tools (Sun *et al.* 2020, Matchado *et al.* 2023), Bac2Feature addresses the issue by removing spurious predictions based on phylogenetic distance.

Bac2Feature provides users an easy-to-use web tool (https://www.genome.jp/bac2feature/) to enhance accessibility to trait prediction using 16S rRNA gene sequences. Thanks to high-throughput sequencing and culturing, vast amounts of amplicon sequencing and prokaryotic trait data are now available. However, a critical gap remains in integrating trait data with microbiome analysis, as multiple steps are needed to annotate traits to amplicon sequences, hindering interpretation of sequencing data using the trait-based approach. Applying Bac2Feature to the human gut microbiome demonstrated that the benefits of this trait-based approach are accessible to users in various fields, including microbial ecology and medical research. This emphasizes the importance of making complex microbiome analyses both interpretable and accessible.

In summary, Bac2Feature fills a critical gap in trait-based approaches by providing a unified, evaluated, and accessible interface for trait prediction using 16S rRNA gene sequences. Future updates should aim to increase the variety of the predicted traits. Although the current version of Bac2Feature does not predict nutrient requirement information, future updates should incorporate the prediction of such metabolic capacities. In addition, the Bac2Feature framework can also be applied to predicting other organisms captured by metabarcoding techniques, such as fungi and plankton.

## Supplementary Material

vbaf136_Supplementary_Data

## Data Availability

All code and data used for of the analysis in this article are publicly available at https://github.com/fuyo780/Bac2Feature and https://github.com/fuyo780/Bac2Feature_experiment.
