# Supplementary material for: Bac2Feature: an easy-to-use interface to predict prokaryotic traits from 16S rRNA gene sequences
Source: Bioinform Adv. 2025 Jun 14;6(1):vbaf136. doi: 10.1093/bioadv/vbaf136 (PMC13770476; doi:10.1093/bioadv/vbaf136)
Supplement: Supplementary file 1 [file vbaf136_supplementary_data.docx]

**Supplements**

**Figure S1.** Description of the trait dataset used in Bac2Feature. Distributions of continuous traits are represented by histograms with fifteen bins, while those of categorical traits are represented by bar plots for each class. The number displayed in the upper right corner of each histogram or bar plot indicates the total count of the data records. Although multi-categorical traits are visualized together for clarity, each is analyzed as a binary trait within the dataset.

 **Figure S2.** Prediction accuracy of the homology-based prediction among variations of homology search methods: BLAST+ (green), VSEARCH (orange), and SortMeRNA (blue). (a) Pearson correlation coefficients of predicted and actual trait values in 10-fold cross-validation for 11 continuous traits. (b) Matthews correlation coefficients in 10-fold cross-validation for 16 categorical traits. Significant differences were detected by Wilcoxon-sighed rank test modified by Benjamini-Hochberg’s method (*q* = 0.05).

 **Figure S3.** Prediction accuracy of the taxonomy-based prediction among variations of taxonomic classification methods: naïve Bayes machine-learning classifier (green), alignment-based taxonomy consensus method based on BLAST+ (orange), and VSEARCH (blue). (a) Pearson correlation coefficients of predicted and actual trait values in 10-fold cross-validation for 11 continuous traits. (b) Matthews correlation coefficients in 10-fold cross-validation for 16 categorical traits. Significant differences were detected by Wilcoxon-sighed rank test modified by the Benjamini-Hochberg’s method (*q* = 0.05).

**Figure S4.** Prediction accuracy of hidden-state-prediction algorithms in the phylogeny-based prediction along with phylogenetic distance for (a) continuous traits and (b) categorical traits. WSCP: weighted squared change parsimony, SA: subtree averaging, PIC: phylogenetic independent contrast, EMP: empirical probability, Mk-SYM: Mk model with symmetrical transition rates, Mk-ARD: Mk model with all different transition rates, MP-no-edge: maximum parsimony without considering edge lengths, MP-edge: maximum parsimony with considering edge lengths. All algorithms were implemented in the R package *castor* (Louca and Doebeli, 2018).

**Figure S5.** Performance evaluation of three representative prediction methods. (a) Precision, (b) recall (true positive rate), and (c) specificity (true negative rate) of the homology, taxonomy, and phylogeny-based prediction in 10-fold cross-validation for 16 categorical traits. Heatmaps on the right show the ranks of the medians of the prediction accuracy of the prediction methods. “Undetermined” means the ranks cannot be determined since statistical difference was detected only between two methods. Entries are sorted starting with the lowest values for the average of prediction accuracy on top.

**Figure S6.** Prediction accuracy of the phylogeny-based prediction in 10-fold cross-validation when using variable regions of 16S rRNA gene sequences for (a) 11 continuous traits and (b) 16 categorical traits.

 **Figure S7.** Cross-validation of the phylogeny-based prediction by non-random sampling based on taxonomic clades at the order, family, and genus levels for (a) continuous trait and (b) categorical traits. The prediction accuracy from the 10-fold cross-validation is shown for comparison. Note that accuracy is reported when the test data included at least one positive example in a given fold; thus, the number of data points in each boxplot may range from 0 to 10. An “X” indicates that no positive examples were present among the test species in that cross-validation fold. Details of the non-random sampling procedure are provided in the Materials and Methods section.

**Figure S8.** Phylogenetic distribution of respiration traits in the 16S rRNA gene phylogenetic tree. Left band shows prokaryotic phylum, while right four bands show the status of respiration traits.

**Table S1.** The rules of our character coding for the categorical traits in the datasets.

| **Trait** | **Initial class** | **Reorganized class** |
| --- | --- | --- |
| Respiration | Obligate aerobic | Aerobic |
| Respiration | Obligate anaerobic | Anaerobic |
| Respiration | Microaerophilic | Aerobic |
| Molility | Flagella | Yes |
| Molility | Gliding | Yes |
| Molility | Axial filament | Yes |
| Temperature range | Psychrotolerant | Psychrophilic |
| Temperature range | Extreme thermophilic | Thermophilic |
| Temperature range | Thermotolerant | Thermophilic |
| Salinity range | Moderate halophilic | Halophilic |
| Salinity range | Halotolerant | Halophilic |
| Salinity range | Extreme halophilic | Halophilic |

**Table S2.** The excluded classes in the multi-categorical traits and their number of data records.

| Class | Multi-categorical traits | Number of data records |
| --- | --- | --- |
| Euryhaline | Salinity range | 1 (0.27%) |
| Stenohaline | Salinity range | 1 (0.27%) |
| Pleomorphic | Cell shape | 32 (0.50%) |
| Disc | Cell shape | 6 (0.093%) |
| Fusiform | Cell shape | 3 (0.047%) |
| Star | Cell shape | 3 (0.047%) |
| Irregular | Cell shape | 2 (0.031%) |
| Square | Cell shape | 1 (0.016%) |
| Spindle | Cell shape | 1 (0.016%) |
| Branced | Cell shape | 1 (0.016%) |

**Table S3.** The number of records in major phyla in the trait dataset used in this study.

| **Phylum** | **Cell diameter** | **Cell length** | **Doubling time** | **Growth temp.** | **Optimum temp.** | **Optimum pH** | **Genome size** | **GC content** | **Coding genes** | **rRNA16S genes** | **tRNA genes** |
| --- | --- | --- | --- | --- | --- | --- | --- | --- | --- | --- | --- |
| **Proteobacteria** | 2039 | 1949 | 223 | 3707 | 2193 | 1359 | 2993 | 1605 | 1103 | 960 | 1093 |
| **Firmicutes** | 1305 | 1243 | 208 | 2093 | 1191 | 707 | 1577 | 795 | 456 | 395 | 446 |
| **Actinobacteria** | 775 | 628 | 25 | 3037 | 1156 | 659 | 1478 | 719 | 356 | 316 | 352 |
| **Bacteroidetes** | 787 | 771 | 16 | 733 | 801 | 517 | 850 | 318 | 160 | 135 | 157 |
| **Euryarchaeota** | 255 | 238 | 171 | 295 | 293 | 243 | 293 | 152 | 118 | 115 | 118 |
| **Tenericutes** | 51 | 9 | 7 | 180 | 52 | 6 | 140 | 96 | 84 | 64 | 81 |
| **Spirochaetes** | 40 | 32 | 17 | 78 | 42 | 25 | 70 | 45 | 34 | 33 | 33 |
| **Deinococcus-Thermus** | 36 | 30 | 12 | 74 | 61 | 38 | 53 | 33 | 23 | 22 | 23 |
| **Crenarchaeota** | 17 | 11 | 34 | 46 | 35 | 15 | 38 | 35 | 31 | 20 | 31 |
| **Thermotogae** | 15 | 15 | 29 | 41 | 30 | 17 | 28 | 18 | 16 | 15 | 16 |
| **Others** | 187 | 163 | 77 | 296 | 229 | 129 | 359 | 253 | 156 | 131 | 150 |
| **All** | 5507 | 5089 | 819 | 10580 | 6083 | 3715 | 7879 | 4069 | 2537 | 2206 | 2500 |

| **Phylum** | **Gram stain** | **Sporulation** | **Motility** | **Salinity range** | **Respiration** | **Temperature range** | **Cell shape** | **All** |
| --- | --- | --- | --- | --- | --- | --- | --- | --- |
| **Proteobacteria** | 3516 | 1909 | 2378 | 206 | 3457 | 842 | 2472 | 34004 |
| **Firmicutes** | 1775 | 1306 | 1228 | 89 | 1863 | 490 | 1332 | 18499 |
| **Actinobacteria** | 2313 | 1130 | 1148 | 55 | 1895 | 448 | 1089 | 17579 |
| **Bacteroidetes** | 799 | 602 | 771 | 21 | 1001 | 182 | 805 | 9426 |
| **Euryarchaeota** | 302 | 108 | 240 | 38 | 352 | 80 | 232 | 3643 |
| **Tenericutes** | 191 | 60 | 43 | 12 | 100 | 41 | 58 | 1275 |
| **Spirochaetes** | 81 | 56 | 59 | 8 | 77 | 31 | 61 | 822 |
| **Deinococcus-Thermus** | 70 | 53 | 63 | 2 | 58 | 23 | 65 | 739 |
| **Crenarchaeota** | 45 | 24 | 23 | 5 | 47 | 25 | 33 | 515 |
| **Thermotogae** | 36 | 20 | 25 | 1 | 36 | 11 | 29 | 398 |
| **Others** | 285 | 205 | 223 | 22 | 272 | 145 | 231 | 3513 |
| **All** | 9413 | 5473 | 6201 | 459 | 9158 | 2318 | 6407 | 90413 |
